# Functional analysis of human mutations in homeodomain transcription factor *PITX3*

**DOI:** 10.1186/1471-2199-8-84

**Published:** 2007-09-21

**Authors:** Satoru Sakazume, Elena Sorokina, Yoshiki Iwamoto, Elena V Semina

**Affiliations:** 1 Department of Pediatrics, Medical College of Wisconsin, 8701 Watertown Plank Road, Milwaukee, WI 53226, USA; 2 Human and Molecular Genetics Center, Medical College of Wisconsin, 8701 Watertown Plank Road, Milwaukee, WI 53226, USA; 3 Division of Clinical Genetics, Gunma Children's Medical Center, Shibukawa, Gunma, Japan; 4 Children's Research Institute, Children's Hospital of Wisconsin and Medical College of Wisconsin, 8701 Watertown Plank Road, Milwaukee, WI 53226, USA; 5 Department of Urology, Medical College of Wisconsin, 8701 Watertown Plank Road, Milwaukee, WI 53226, USA; 6 Department of Surgical Research, Beckman Research Institute of the City of Hope, Duarte, CA 91010-3000, USA

## Abstract

**Background:**

The homeodomain-containing transcription factor *PITX3 *was shown to be essential for normal eye development in vertebrates. Human patients with point mutations in *PITX3 *demonstrate congenital cataracts along with anterior segment defects in some cases when one allele is affected and microphthalmia with brain malformations when both copies are mutated. The functional consequences of these human mutations remain unknown.

**Results:**

We studied the PITX3 mutant proteins S13N and G219fs to determine the type and severity of functional defects. Our results demonstrate alterations in DNA-binding profiles and/or transactivation activities and suggest a partial loss-of-function in both mutants with the G219fs form being more severely affected. No anomalies in cellular distribution and no dominant-negative effects were discovered for these mutants. Interestingly, the impairment of the G219fs activity varied between different ocular cell lines.

**Conclusion:**

The G219fs mutation was found in multiple families affected with congenital cataracts along with anterior segment malformations in many members. Our data suggest that the presence/severity of anterior segment defects in families affected with G219fs may be determined by secondary factors that are expressed in the developing anterior segment structures and may modify the effect(s) of this mutation. The S13N mutant showed only minor alteration of transactivation ability and DNA binding pattern and may represent a rare polymorphism in the *PITX3 *gene. A possible contribution of this mutation to human disease needs to be further investigated.

## Background

Cataracts remain a leading cause of blindness worldwide accounting for 42% of all blindness [1,2]. In one large study, about 30% of all congenital/infantile cataracts was attributed to genetic factors as identified by the presence of multiple affected family members or the association of other dysmorphology suggestive of a genetic syndrome [3]. The etiology of about 87% of unilateral and 50% of bilateral cataracts remains unknown [3], and is likely to have some genetic basis as well. In recent years, multiple genes underlying human congenital cataracts have been identified and opened the way to functional studies and a better understanding of lens development [4].

*PITX3 *is a homeodomain transcription factor found to be responsible for human congenital cataract that can be associated with abnormal development of the anterior segment. *PITX3 *belongs to the *paired*-like group and a *bicoid*-like subgroup. The *bicoid*-like homeodomains are characterized by a lysine at position 50 (position 9 of the third helix) in the homeodomain, which is known to selectively recognize the 3' CC dinucleotide adjacent to the TAAT core sequence [5-8].

The function of the *PITX3 *gene in ocular development is highly conserved in vertebrates. The mouse and rat *Pitx3 *genes were identified in 1997 and demonstrate strong expression during lens and brain development [9,10]. The mouse *Pitx3 *gene was shown to be involved in *aphakia*, a recessive mutant phenotype characterized by small eyes lacking the lens [11-13]. Recent publications demonstrated that *Xenopus *and *Danio rerio pitx3 *genes are highly similar in sequence, expression, and function to their mammalian homologs [14-16]. Morpholino-induced knockdown of *pitx3 *at early embryonic stages in zebrafish resulted in a lens and retinal phenotype similar to the one seen in the *aphakia *mouse mutant [16-18].

Originally, two different *PITX3 *mutations were reported in human patients [19]. The first mutation was a C-terminal 17-bp insertion that resulted in a frameshift and abnormal configuration of ~1/3 of the protein (G219fs); this mutation was found in a multigenerational family with anterior segment ocular dysgenesis and cortical cataracts [19,20]. The second mutation was a serine to asparagine substitution in the N-terminal region of the protein [S13N]; this mutation was found in a mother and child affected with congenital cataract. Several recent publications reported a recurrence of the same 17-bp insertion mutation in seven families of different ethnic backgrounds affected with congenital posterior polar cataract that, in some cases, included anterior segment defects [21-25]. An additional C-terminal single-nucleotide deletion, G217fs, was identified in two families affected with posterior polar cataract; this mutation is predicted to result in a truncation of the normal protein sequence around the same site (only two amino acids upstream) as the recurrent 17-bp insertion [21,24]. Interestingly, in one family two siblings from a consanguineous marriage were found to be homozygous for the mutation and demonstrated microphthalmia and central nervous system defects [24]. To date, no mutations in the homeodomain region of *PITX3 *have been identified.

Distribution of *PITX3 *mutations is remarkably different from that seen in other homeodomain proteins including its close family member, *PITX2*. *PITX2 *mutations are clustered in the homeodomain region with several C-terminal and no N-terminal mutations identified to date [26,27]. The majority of *PITX2 *mutations were shown to result in a complete loss-of-function with a few partial loss-of-function, one gain-of-function and one dominant-negative mutations being reported as well [6,28-34].

In contrast to *PITX2 *mutations, the homeodomain region remains intact in *PITX3 *mutants identified thus far, while the C-terminal or N-terminal regions are affected. The mechanism(s) by which these mutations obstruct normal function of *PITX3 *have never been investigated. The N-terminal and C-terminal regions of PITX1&2 proteins have been implicated into protein-protein interactions that affect PITX protein binding/activation of target promoters [28,35,36]. Therefore *PITX3 *mutations may be highly susceptible to modulation by secondary factors and express variable phenotypes depending on the genetic background. Alternatively, mutations in non-DNA-binding domains of this transcription factor may lead to mutant proteins that are dominant-negative to wild-type activity. Characterization of *PITX3 *mutations is important for better understanding of the human phenotypes associated with alterations in this gene.

## Results

### Analysis of PITX3 normal sequence and mutations

**Figure 1:**
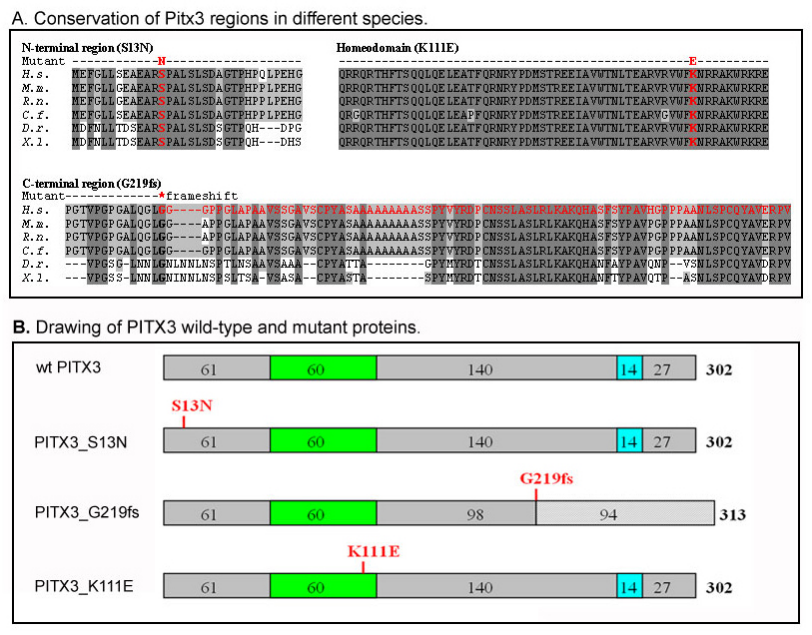
**PITX3 wild-type and mutant proteins**. **A**. Conservation of Pitx3 regions in different species. Amino acids that are identical in all or at least four proteins are highlighted in dark and light grey, correspondingly. Positions of mutations are shown in red. *H.s- Homo sapiens, M.m.- Mus musculus, R.n.- Rattus Norvegicus, C.f.- Canis familiaris, D.r.- Danio rerio, X.l.- Xenopus laevis*. **B**. Drawing of PITX3 wild-type and mutant proteins. The homeodomain is shown as green box, the OAR domain is shown as blue box and positions of mutations are indicated in red.

The *PITX3 *mutations identified thus far are represented by alterations of the N- or C-terminal domains (Table 1). We analyzed PITX3 predicted protein sequences in different species for conservation at the mutant nucleotide positions (Figure 1A). The S13 position was found to be 100% conserved in all known Pitx3 proteins from humans to zebrafish while the eighty-three C-terminal amino acids that are missing in the G219fs mutant demonstrate ~60% identity between different species. This analysis suggested important roles for both regions in normal protein function. For control, an additional *PITX3 *mutant was included in our study, K111E, which was constructed to carry a mutation at position 50 of the homeodomain that changes a lysine into glutamic acid. The K111E alteration is identical to the K88E mutation in PITX2 homeodomain that was shown to have severe DNA-binding and transactivation defects and also demonstrated a dominant-negative effect [33,34].

**Table 1:** Human disorders associated with *PITX3 *mutations

**PITX3 genotype**	**Protein defect**	**Human ocular phenotype**	**# independent reports**	**Reference**
WT/G38A	S13N (N-term)	congenital total cataract with glaucoma	1	19
WT/650delG	G217fs (C-term)	congenital posterior polar cataract	2	21; 24
650delG/650delG	G217fs (C-term)	microphthalmia	1	24
WT/657ins17	G219fs (C-term)	congenital posterior polar cataract	4	21–23; 25
		congenital posterior polar cataract with anterior segment dysgenesis	2	21
		congenital cortical cataract with anterior segment dysgenesis	1	19

These *PITX3 *sequences encoding wild-type and mutant proteins were inserted into the pcDNA3.1 expression plasmid in frame with *myc *epitope (Figure 1B), transfected into B3 lens epithelial cells and analyzed for expression by Western blots. As expected, a ~37 kDa band was detected for the wild-type, S13N and K111E forms and a slightly larger product was observed in cells transfected with the G219fs- expressing plasmid. The wild-type and mutant recombinant proteins were examined in respect to their cellular localization, DNA-binding and transactivation capabilities as well as potential dominant-negative effects.

### Analysis of cellular distribution of PITX3 wild-type and mutant proteins

**Figure 2:**
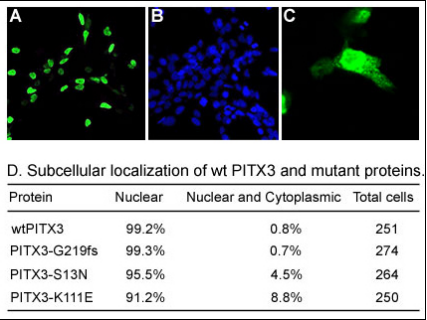
**Subcellular localization of PITX3 wild-type and mutant proteins**. **A**. Image of a slide that was stained with *a myc *antibody that recognizes recombinant PITX3. **B**. Image of a slide that was stained with DAPI. **C**. Example of detection of nuclear and cytoplasmic localization.**D**. Summary of cell counts for nuclear and cytoplasmic localization for different PITX3 forms.

In order to analyze cellular distribution of wild-type and mutant PITX3 proteins, expression plasmids were transfected into B3 lens epithelial cells and then the cells were collected for immunostaining with *myc *antibody (Figure 2). The transfected cells were examined in four low power fields; the numbers of positive cells varied from 250 to 274 (~30% transfection efficiency). Consistent with its role as a transcription factor, 99% of the wild-type PITX3 protein was localized to the nucleus. The PITX3 mutant forms demonstrated similar distribution with 99% of G219fs, 96% of S13N and 91% of K111E proteins being found exclusively in the nucleus and the remainder showing both cytoplasmic and nuclear staining (Figure 2).

### Analysis of DNA-binding patterns of PITX3 wild-type and mutant proteins

We analyzed the DNA-binding properties of wild-type, S13N and G219fs mutant PITX3 proteins by electrophoretic mobility shift assay (EMSA). Some previously reported studies demonstrated that, similar to other PITX factors, mouse Pitx3 is able to bind to and transactivate promoters containing *bicoid *(TAATCC) sequences (tyrosine hydroxylase promoter; 7). Therefore an oligonucleotide containing two *bicoid *sites [34] was employed for EMSA. The *bicoid*-like sequences TAAGCT and AAAGCC were also shown to bind Pitx3 but were not examined in our study [7,8].

**Figure 3:**
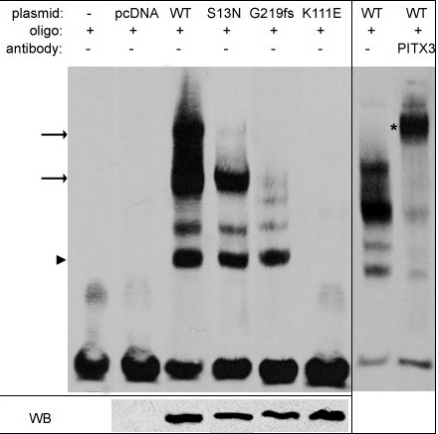
**DNA-binding patterns of PITX3 wild-type and mutant proteins**. EMSA (left panel) showed alterations in the binding patterns of S13N, G219fs and K111E proteins in comparison to wild-type. The normal pattern of WT PITX3-DNA binding consisted of several bands with the low mobility complexes likely representing homo- or hetero- dimer DNA interactions (black arrows) and the fast moving band corresponding to PITX3 monomers binding DNA (black arrowhead); all complexes disappeared after addition of anti-PITX3 antibody to reaction (right panel; supershift band is indicated with black asterisk). No DNA-protein complexes were observed for K111E mutant and some low mobility complexes (indicated by black arrows) were not present in EMSAs performed with S13N or G219fs mutant extracts. Western blot (bottom) confirms the presence of PITX3 forms in the corresponding cells.

EMSA assays demonstrated that wild-type PITX3 protein forms DNA-protein complexes with the *bicoid *probe as expected. The pattern consisted of several bands of different electrophoretic mobility (Figure 3). The B3 lens epithelial cell extracts obtained after transfection with an empty pcDNA3.1 plasmid were used to confirm that the *bicoid*-probe binding activity is not a property of B3 cells or vector sequences. Addition of an antibody against human PITX3 resulted in the formation of a supershift and disappearance of all complexes (Figure 3). The fastest-migrating EMSA band probably corresponds to PITX3 binding DNA as a monomer and the slower moving bands may represent PITX3 homodimer- or PITX3 heterodimer- DNA complexes with other proteins. Multiple homeodomain proteins were shown to bind DNA as monomers and homodimers [34,37-39] or in association with other proteins [40,41].

Both the S13N and G219fs mutant proteins appear to retain their ability to bind DNA as monomers (Figure 3); however, formation of lower mobility complexes was affected to varying degrees. The DNA-binding profile of the S13N mutant was comparable to wild-type protein with only the largest multiprotein-DNA complex being affected. For the G219fs protein, the two main low electrophoretic mobility complexes were found to be absent (Figure 3). The K111E mutant showed no binding to the *bicoid *probes, as expected. Equivalent amounts of the *myc*-tagged PITX3 proteins were present in the nuclear extracts that were used in these experiments.

### Analysis of transactivation activity of PITX3 wild-type and mutant forms in lens epithelial and corneal stromal cells

To analyze the transactivation activities of the PITX3 wild-type and mutant forms, we employed the *bicoid*-TK-luc target promoter that has been previously used to characterize activities of another member of this family, PITX2 [6].

**Figure 4:**
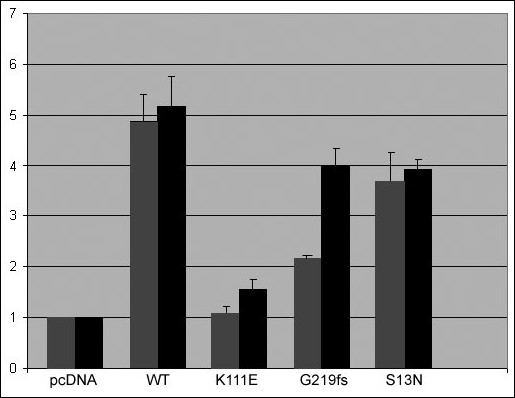
**Transactivation of *bicoid*-Tk-luc reporter in lens and corneal cells by wild-type and mutant PITX3**. After normalization to β-galactosidase activity, the data are presented as luciferase values observed in co-transfections of *bicoid*-Tk-luc with various PITX3 forms relative to the activity of same reporter with empty vector only (pcDNA3.1). Values observed in transient transfections into B3 lens epithelial cell line are shown in grey and corneal stromal cells in black; error bars that denote SD are indicated.

When the wild-type *PITX3 *was co-transfected with the *bicoid*-TK-luc plasmid into B3 lens epithelial or corneal stromal cells, a ~5-fold increase in reporter gene activity was consistently observed (Figure 4). When *PITX3 *mutants *S13N *and *G219fs *were co-transfected with the same reporter, the increase in reporter activity was found to be 77% and 46% of the wild-type level in lens epithelial cells and at 76% and 78% in corneal stromal cells, respectively (Figure 4). In contrast, when the *K111E *mutant was co-transfected with the target promoter, no activation was observed in lens cells and only a slight up-regulation was detected in corneal cells.

**Figure 5:**
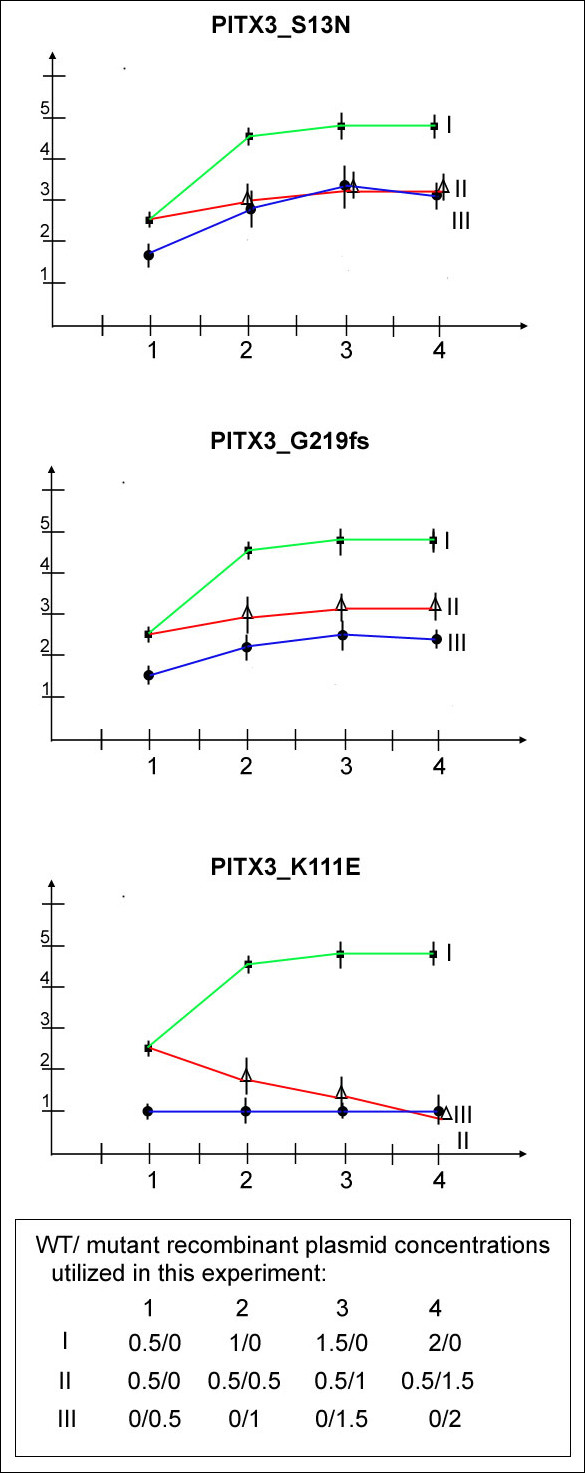
**Examination of PITX3 mutant forms for dominant-negative effect**. The data are presented as luciferase values observed in co-transfections of the reporter with the PITX3 plasmids relative to the activity of same reporter with empty pcDNA3.1 vector only; all experiments were performed in B3 lens epithelial cells. Data for S13N (top), G219fs (middle) and K111E mutants (bottom) are shown. The green line connects values observed in transient co-transfections of 0.5-, 1-, 1.5- and 2-μg of wild-type PITX3 and the reporter (1 μg). The blue line connects values observed in transient co-transfections of 0.5-, 1-, 1.5- and 2-μg of mutant PITX3 with the reporter (1 μg). The values observed in experiments when the reporter (1 μg) was co-transfected with 0.5 μg of wild-type PITX3 together with increasing amounts of S13N, G219fs or K111E mutants (0.5-, 1- and 1.5-μg) are joined by a red line.

To examine whether the mutant proteins exert a dominant-negative effect, the *bicoid*-TK-luc reporter, wild-type and mutant forms were co-transfected into lens epithelial cells. In these assays, the amount of wild type *PITX3 *plasmid was kept at 0.5 μg and *bicoid*-TK-luc reporter at 1 μg while varied amounts of mutant constructs (from 0.5 μg to 1.5 μg) were added. To ensure that the total amount of DNA was equivalent in all assays, some reactions were adjusted with pcDNA3.1 vector DNA. No dominant-negative activity was detected for either the *S13N *or *G219fs *mutants while the control *K111E *mutant demonstrated a dominant-negative effect as expected (see above; Figure 5).

## Discussion

PITX3 represents a homeodomain-containing transcription factor that was shown to be essential to normal embryonic eye and brain development in vertebrates. Identified disease-causing human *PITX3 *mutations are represented by three sequence alterations, one affecting the coding region for the N-terminal and the other two affecting the C-terminal regions of the protein. No mutations in the region encoding the homeodomain, a common site for pathologic mutations in this protein class, have been reported. The *PITX3-S13N *(N-terminal) mutation was identified in a family with autosomal-dominant congenital total cataract while the recurrent C-terminal mutation, *PITX3-G219fs*, was found in multiple pedigrees affected with dominant forms of cataracts often accompanied by severe anterior segment dysgenesis.

The N-terminal *S13N *mutation appears to be located within a conserved stretch of 11-amino acids, EARSPALSLSD. This motif seems to be specific to the PITX3 family and was not found in any other PITX proteins. The function of this motif and, in fact, the entire PITX3 N-terminal arm is not clear at this point although, in general, N-terminal regions of homeodomain proteins were shown to be involved in DNA-binding, dimerization, phosphorylation and interaction with cofactors [42-45]. The C-terminal *G219fs *mutation results in a truncation of the normal PITX3 sequence at ~2/3 of its normal length and inclusion of ninety-four additional erroneous residues. The region around the OAR domain and the last twelve residues appear to be most conserved; the OAR domain represents a stretch of 14-amino-acids that were found to be identical in various *paired*-like homeodomain proteins [19,46,47]. The functions of these domains are not fully determined yet; studies of Cart1 and Prx2, other OAR-containing proteins, showed that the OAR domain seems to perform an inhibitory function as deletion of the domain resulted in increased DNA binding or transactivation of target promoters [48,49]. The C-terminal parts of other PITX proteins were shown to play multiple regulatory roles and be involved in specific protein-protein interactions with such factors as Pit-1 or Lef-1 [28,50]. In summary, the studied mutations affect the configuration of different conserved regions of the PITX3 protein. Since these regions are involved in various and yet to be determined interactions, the functional consequences of the N- and C-terminal mutations may be different and therefore provide diverse mechanisms for associated ocular defects.

Both the S13N and G219fs mutant proteins as well as the K111E control mutant were found to be expressed and to localize to the nucleus, similar to wild-type PITX3. The K111E protein was found to have the highest incidence of cytoplasmic occurrence, which may be due to K111E interference with a likely nuclear localization signal located one amino acid downstream from the affected site (see Figure 1A). The RRAKWRK sequence in the third helix of the PITX3 homeodomain is similar to the sequences identified in several other homeodomain proteins as necessary and, in conjunction with the integrity of the helix 3 domain, sufficient for the nuclear transport of PDX-1, PITX2 and SHOX [51-55].

Defective functions of the S13N and G219fs mutant proteins were revealed in electrophoretic mobility shift assays and transactivation activity assays. The normal pattern of wild-type PITX3- DNA binding consisted of several bands with the low mobility complexes likely representing homo- or hetero- dimer DNA interactions and the fast moving band corresponding to PITX3 monomers binding DNA; similar patterns were reported for the PITX2 protein [34]. EMSA demonstrated that both mutants form high mobility complexes of similar intensity to wild-type protein and therefore appear to retain the ability to bind DNA as monomers. At the same time, the formation of lower mobility complexes was somewhat disturbed for S13N and significantly affected for the G219fs mutant. Therefore the ability of mutants to form these complexes and/or to bind DNA as homo- or hetero-dimers seems to be damaged. The severe alteration in formation of higher order protein-protein DNA-binding complexes by the G219fs mutant is not surprising since the PITX C-terminal region was shown to play an important role in dimerization and interactions with other proteins [34].

Both the S13N and G219fs mutants demonstrated a decrease in the transactivation activity of a promoter containing *bicoid *elements in lens epithelial and corneal stromal cell lines. Interestingly, the transactivation activity of the mutant proteins was higher in corneal cells in comparison to lens cells with the G219fs mutant demonstrating the most remarkable difference, 46% and 78% of WT activity in lens and corneal cells, respectively. The studied S13N and G219fs proteins contain normal homeodomain sequence and, as demonstrated by EMSA, are still capable of interacting with DNA. Therefore, it is possible that these mutations may act in a dominant-negative fashion by blocking wild-type proteins from efficient binding to *bicoid *sites. Another possibility would be that these mutants may make inactive dimers with wild-type protein and thus impair wild-type protein function [[33,34] and [56]]. No dominant-negative effect was evident in co-transfection assays of mutant and wild-type PITX3 forms.

The S13N form demonstrated only minor functional alterations in comparison to G219fs mutant. This is consistent with the fact that this is a relatively conservative amino acid transition (both the serine and asparagine residues are uncharged under physiologic conditions). It should also be taken in consideration that the *G219fs *mutation was found in several unrelated large pedigrees affected with congenital posterior polar cataract while the S13N change was only identified in a single pedigree consisting of a mother and child affected with congenital total cataracts. Our functional studies discovered only mild alterations of S13N function in comparison to wild-type. Therefore it is possible that *S13N *represents a rare variant in the *PITX3 *gene that is contributing to, but not causing, the disease in this family. At the same time, further studies into PITX3 function(s) are required to be able to fully explore the properties of this mutant and its possible involvement in human disease.

The variability of the phenotype associated with the *G219fs *mutation may be explained by the contribution of secondary factors modifying the ability of this mutant to transactivate target promoters. This effect was evident in human corneal cells transfected with the *G219f*s-mutant/*bicoid*-reporter when a significant rescue of transactivation activity was observed in comparison to experiments performed in human lens cells. It is plausible to suggest that, in some patients, certain allelic combination of interacting factors is capable of rescuing G219fs transactivation activity so that levels of functional PITX3 reach a threshold required for normal development of the anterior segment structures while in other patients this function can not be restored and therefore results in various abnormalities of the anterior segment.

## Conclusion

Variable alterations in the DNA-binding profile and transactivation activity were identified in two PITX3 mutant forms associated with ocular phenotypes, S13N and G219fs. Mutant transactivation activity was found to be variable between human lens and corneal cells suggesting the presence of secondary factors able to modify/rescue mutant protein function. The observed partial rescue of mutant activity in corneal cells may provide an explanation for the fact that only a subset of human patients with *PITX3 *mutations demonstrate anterior segment/corneal anomalies while cataract/lens defects are present in all cases. Identification of secondary factors that modify PITX *3 *function in developing corneal tissues may facilitate better prediction of ocular features in affected individuals. Corneal abnormalities represent the most challenging feature to treat in these patients and identification of modifying factors will lead to better understanding of the underlying mechanism(s). In addition, since the studied N- and C-terminal *PITX3 *mutant forms demonstrated only partial loss-of-function, the lack of mutations in the *PITX3 *homeodomain region in families with cataract/anterior segment ocular dysgenesis may be due to the fact that these types of mutations result in a more severe alteration of PITX3 function and therefore produce a more pronounced phenotype or result in prenatal death.

## Methods

### DNA constructs

The human *PITX3*, *PITX3_S13N*, and *PITX3_G219fs *cDNAs were cloned into the pcDNA3.1 MycHisC expression vector containing the T7 promoter and an in-frame C-terminal c-Myc epitope (Invitrogen). *PITX3_K111E *was created using the Gene Editor site-directed mutagenesis kit (Promega). The *bicoid*-Tk-luciferase reporter contains the herpes simplex thymidine kinase minimal promoter with four *bicoid *elements [6,33,34].

### Cell culture, transfection and luciferase assays

B3 human lens epithelial cells were obtained from ATCC (CRL-11421™) and cultured in medium as suggested by the supplier. Human corneal stromal cells were a generous gift of Dr. Watsky (University of Tennessee) [57] these cells were propagated in the DMEM medium containing 10% FBS. We examined human lens and corneal cells for expression of endogenous PITX3 and compared it with expression of our recombinant proteins: in lens epithelial cells, endogenous PITX3 protein represented 1/55- 1/100 fraction of recombinant proteins while no endogenous PITX3 protein was detected in human corneal cells by Western blotting (low levels of PITX3 transcript were identified by RT-PCR). Densitometry analysis was performed on a Macintosh computer using the public domain NIH Image program [58]. The B3 human lens epithelial cells and human corneal stromal cells were cultured in 6-well tissue culture plates. In transfection assays, the manufacturer's protocols were followed (Invitrogen); in short, 1.5 μg of reporter DNA, 1.5 μg of effectors DNA, 0.5 μg of pcDNA_lacZ (for normalization of transfection efficiency), 1.75 μl of reagent Plus and 5.25 μl of LipofectamineLTX or 2000 (Invitrogen) were added to every well in 0.5 ml of OPTI-MEM medium. When B3 lens epithelial cells were grown in 24-well plates, the cells were transfected using 1/5 of the volume of transfection mixture for 6-well plates. The cells were collected 24 hours after transfection and assayed for luciferase using luciferase assay system (Promega). β-galactosidase activity was measured in lysates using β-Galactosidase Assay System (Promega). Each experiment was performed in three replicates and transfections were independently repeated at least three times.

### Electrophoretic Mobility Shift Assay

Oligonucleotide, 5'GATCCTAATCCCGTCGCGTCGTAATCCGGATC3' containing two *bicoid *sites separated by 10 nucleotides (Bcd 2x 10n; [34]) was used in this study. For oligonucleotide labeling and detection, Biotin 3' End DNA Labeling Kit (Pierce) and LightShift Chemiluminescent EMSA Kit (Pierce) were used. Nuclear extracts were prepared from B3 cells transiently transfected with the corresponding plasmid. Nuclear proteins were released by high-salt extraction buffer after the cytoplasmic fraction was removed using CelLytic NuCLEAR extraction kit (Sigma). Recombinant protein concentrations in each extract were estimated by Western blot and densitometry. The amount of extract for each binding reaction was normalized by recombinant protein concentration and the difference in salt concentration was adjusted with extraction buffer. 20 fmol of biotin end-labeled probe were mixed with binding buffer containing 50 ng/μl of Poly [d(I-C)]. Binding reactions were incubated at RT for 15 minutes. For supershift, 1 μg of goat polyclonal Pitx3 antibody (Santa-Cruz) or 1 μg of mouse monoclonal Myc-Tag (9B11) antibody (Cell Signalling) was added to the binding reaction mix, and incubation was extended to 30 minutes at RT. Electrophoresis was performed using 10-cm long 1× TBE 5% polyacrylamide gels for one hour at 80 V; the gels were then blotted onto positively charged nylon membrane (Roche) for 30 minutes at 400 mV with 1× TBE.

### Western blot and immunocytochemistry

For Western blot, 2 μg of whole cell extract from transfected cells were electrophoresed into 10% SDS-polyacrylamide gel, transferred to polyvinylidine difluoride filters (Millipore), and immunoblotted using Myc-Tag (9B11) Monoclonal antibody (Cell Signaling). After reaction with a secondary antibody conjugated with HRP, signal was detected with PIERCE ECL Western Blotting Substrate. For reaction immunocytochemistry, B3 lens epithelial cells were cultured on cover slips until 50 to 80% confluency. After 4% paraformaldehyde fixation, 9B11 Myc-tag antibody (Cell Signaling) and FITC-conjugated Goat Anti-Mouse IgG were added for detection of positive cells. VectaShield with DAPI (Vector Laboratories) was used to antifade mounting medium. For observation of positively stained cells, Nikon Ecripse600 Fluoroscope was utilized.

## Abbreviations

EMSA, electrophoretic mobility shift assay; DAPI, 4',6-Diamidino-2-phenylindole; HD, homeodomain; OAR-domain, *otp, aristaless, and rax *domain; ASMD- anterior segment mesenchymal dysgenesis.

## Competing interests

The author(s) have declared that there are no competing interests.

## Authors' contributions

SS was responsible for generation of constructs, immunocytochemistry, transfection experiments and dominant-negative assays and helped to draft the manuscript; ES performed transfection experiments and gel shift assays and helped to draft the manuscript; YI was involved in designing the study and helped to draft the manuscript; EVS conceived and supervised the study, and drafted the manuscript. All authors have read and approved the final manuscript.

## References

[B1] Thylefors B, Négrel AD, Pararajasegaram R, Dadzie KY (1995). Global data on blindness. Bull World Health Organ.

[B2] Thylefors B (1998). A global initiative for the elimination of avoidable blindness. Am J Ophthalmol.

[B3] Haargaard B, Wohlfahrt J, Fledelius HC, Rosenberg T, Melbye M (2004). A nationwide Danish study of 1027 cases of congenital/infantile cataracts: etiological and clinical classifications. Ophthalmology.

[B4] Graw J (2004). Congenital hereditary cataracts. Int J Dev Biol.

[B5] Hanes SD, Brent R (1991). A genetic model for interaction of the homeodomain recognition helix with DNA. Science.

[B6] Amendt BA, Sutherland LB, Semina EV, Russo AF (1998). The molecular basis of Rieger syndrome. Analysis of Pitx2 homeodomain protein activities. J Biol Chem.

[B7] Lebel M, Gauthier Y, Moreau A, Drouin J (2001). Pitx3 activates mouse tyrosine hydroxylase promoter via a high-affinity binding site. J Neurochem.

[B8] Cazorla P, Smidt MP, O'Malley KL, Burbach JP (2000). A response element for the homeodomain transcription factor Ptx3 in the tyrosine hydroxylase gene promoter. J Neurochem.

[B9] Semina EV, Reiter RS, Murray JC (1997). Isolation of a new homeobox gene belonging to the Pitx/Rieg family: expression during lens development and mapping to the aphakia region on mouse chromosome 19. Hum Mol Genet.

[B10] Smidt MP, van Schaick HS, Lanctot C, Tremblay JJ, Cox JJ, van der Kleij AA, Wolterink G, Drouin J, Burbach JP (1997). A homeodomain gene Ptx3 has highly restricted brain expression in mesencephalic dopaminergic neurons. Proc Natl Acad Sci USA.

[B11] Varnum DS, Stevens LC (1969). Aphakia, a new mutation in the mouse. J Hered.

[B12] Semina EV, Murray JC, Reiter R, Hrstka RF, Graw J (2000). Deletion in the promoter region and altered expression of Pitx3 homeobox gene in aphakia mice. Hum Mol Genet.

[B13] Rieger DK, Reichenberger E, McLean W, Sidow A, Olsen BR (2001). A double-deletion mutation in the Pitx3 gene causes arrested lens development in aphakia mice. Genomics.

[B14] Pommereit D, Pieler T, Hollemann T (2001). Xpitx3: a member of the Rieg/Pitx gene family expressed during pituitary and lens formation in Xenopus laevis. Mechanisms of Development.

[B15] Khosrowshahian F, Wolanski M, Chang WY, Fujiki K, Jacobs L, Crawford MJ (2005). Lens and retina formation require expression of Pitx3 in Xenopus pre-lens ectoderm. Dev Dynamics.

[B16] Shi X, Bosenko D, Zinkevich N, Foley S, Hyde DR, Semina EV, Vihtelic TS (2005). Zebrafish pitx3 is necessary for normal lens and retinal development. Mechanisms of Development.

[B17] Dutta S, Dietrich JE, Aspock G, Burdine RD, Schier A, Westerfield M, Varga ZM (2005). pitx3 defines an equivalence domain for lens and anterior pituitary placode. Development.

[B18] Zilinski CA, Shah R, Lane ME, Jamrich M (2005). Modulation of zebrafish pitx3 expression in the primordia of the pituitary, lens, olfactory epithelium and cranial ganglia by hedgehog and nodal signaling. Genesis.

[B19] Semina EV, Ferrell RE, Mintz-Hittner HA, Bitoun P, Alward WL, Reiter RS, Funkhauser C, Daack-Hirsch S, Murray JC (1998). A novel homeobox gene PITX3 is mutated in families with autosomal-dominant cataracts and ASMD. Nat Genet.

[B20] Hittner HM, Kretzer FL, Antoszyk JH, Ferrell RE, Mehta RS (1982). Variable expressivity of autosomal dominant anterior segment mesenchymal dysgenesis in six generations. Am J Ophthalmol.

[B21] Berry V, Yang Z, Addison PK, Francis PJ, Ionides A, Karan G, Jiang L, Lin W, Hu J, Yang R, Moore A, Zhang K, Bhattacharya SS (2004). Recurrent 17 bp duplication in PITX3 is primarily associated with posterior polar cataract (CPP4). J Med Genet.

[B22] Addison PK, Berry V, Ionides AC, Francis PJ, Bhattacharya SS, Moore AT (2005). Posterior polar cataract is the predominant consequence of a recurrent mutation in the PITX3 gene. Br J Ophthalmol.

[B23] Finzi S, Li Y, Mitchell TN, Farr A, Maumenee IH, Sallum JM, Sundin O (2005). Posterior polar cataract: genetic analysis of a large family. Ophthalmic Genet.

[B24] Bidinost C, Matsumoto M, Chung D, Salem N, Zhang K, Stockton DW, Khoury A, Megarbane A, Bejjani BA, Traboulsi EI (2006). Heterozygous and homozygous mutations in PITX3 in a large Lebanese family with posterior polar cataracts and neurodevelopmental abnormalities. Invest Ophthalmol Vis Sci.

[B25] Burdon KP, McKay JD, Wirth MG, Russell-Eggit IM, Bhatti S, Ruddle JB, Dimasi D, Mackey DA, Craig JE (2006). The PITX3 gene in posterior polar congenital cataract in Australia. Mol Vis.

[B26] Strungaru MH, Dinu I, Walter MA (2007). Genotype-phenotype correlations in Axenfeld-Rieger malformation and glaucoma patients with FOXC1 and PITX2 mutations. Invest Ophthalmol Vis Sci.

[B27] Hjalt TA, Semina EV (2005). Current molecular understanding of Axenfeld-Rieger syndrome. Expert Rev Mol Med.

[B28] Amendt BA, Sutherland LB, Russo AF (1999). Multifunctional role of the Pitx2 homeodomain protein C-terminal tail. Mol Cell Biol.

[B29] Espinoza HM, Cox CJ, Semina EV, Amendt BA (2002). A molecular basis for differential developmental anomalies in Axenfeld-Rieger syndrome. Hum Mol Genet.

[B30] Lines MA, Kozlowski K, Kulak SC, Allingham RR, Heon E, Ritch R, Levin AV, Shields MB, Damji KF, Newlin A, Walter MA (2004). Characterization and prevalence of PITX2 microdeletions and mutations in Axenfeld-Rieger malformations. Invest Ophthalmol Vis Sci.

[B31] Perveen R, Lloyd IC, Clayton-Smith J, Churchill A, van Heyningen V, Hanson I, Taylor D, McKeown C, Super M, Kerr B, Winter R, Black GC (2000). Phenotypic variability and asymmetry of Rieger syndrome associated with PITX2 mutations. Invest Ophthalmol Vis Sci.

[B32] Priston M, Kozlowski K, Gill D, Letwin K, Buys Y, Levin AV, Walter MA, Heon E (2001). Functional analyses of two newly identified PITX2 mutants reveal a novel molecular mechanism for Axenfeld-Rieger syndrome. Hum Mol Genet.

[B33] Saadi I, Semina EV, Amendt BA, Harris DJ, Murphy KP, Murray JC, Russo AF (2001). Identification of a dominant negative homeodomain mutation in Rieger syndrome. J Biol Chem.

[B34] Saadi I, Kuburas A, Engle JJ, Russo AF (2003). Dominant negative dimerization of a mutant homeodomain protein in Axenfeld-Rieger syndrome. Mol Cell Biol.

[B35] Bach I, Carriere C, Ostendorff HP, Andersen B, Rosenfeld MG (1997). A family of LIM domain-associated cofactors confer transcriptional synergism between LIM and Otx homeodomain proteins. Genes and Development.

[B36] Kioussi C, Briata P, Baek SH, Rose DW, Hamblet NS, Herman T, Ohgi KA, Lin C, Gleiberman A, Wang J, Brault V, Ruiz-Lozano P, Nguyen HD, Kemler R, Glass CK, Wynshaw-Boris A, Rosenfeld MG (2002). Identification of a Wnt/Dvl/beta-Catenin --> Pitx2 pathway mediating cell-type-specific proliferation during development. Cell.

[B37] Green PD, Hjalt TA, Kirk DE, Sutherland LB, Thomas BL, Sharpe PT, Snead ML, Murray JC, Russo AF, Amendt BA (2001). Antagonistic regulation of Dlx2 expression by PITX2 and Msx2: implications for tooth development. Gene Expr.

[B38] Quirk J, Brown P (2002). Hesx1 homeodomain protein represses transcription as a monomer and antagonises transactivation of specific sites as a homodimer. J Mol Endocrinol.

[B39] Zaffran S, Frasch M (2005). The homeodomain of Tinman mediates homo- and heterodimerization of NK proteins. Biochem Biophys Res Commun.

[B40] Sarno JL, Kliman HJ, Taylor HS (2005). HOXA10, Pbx2, and Meis1 protein expression in the human endometrium: formation of multimeric complexes on HOXA10 target genes. J Clin Endocrinol Metab.

[B41] Bai S, Shi X, Yang X, Cao X (2000). Smad6 as a transcriptional corepressor. J Biol Chem.

[B42] Banham AH, Asante-Owusu RN, Gottgens B, Thompson S, Kingsnorth CS, Mellor E, Casselton LA (1995). An N-Terminal Dimerization Domain Permits Homeodomain Proteins To Choose Compatible Partners and Initiate Sexual Development in the Mushroom Coprinus cinereus. Plant Cell.

[B43] Phelan ML, Featherstone MS (1997). Distinct HOX N-terminal arm residues are responsible for specificity of DNA recognition by HOX monomers and HOX.PBX heterodimers. J Biol Chem.

[B44] Palena CM, Tron AE, Bertoncini CW, Gonzalez DH, Chan RL (2001). Positively charged residues at the N-terminal arm of the homeodomain are required for efficient DNA binding by homeodomain-leucine zipper proteins. J Mol Biol.

[B45] Espinoza HM, Ganga M, Vadlamudi U, Martin DM, Brooks BP, Semina EV, Murray JC, Amendt BA (2005). Protein kinase C phosphorylation modulates N- and C-terminal regulatory activities of the PITX2 homeodomain protein. Biochemistry.

[B46] Semina EV, Reiter R, Leysens NJ, Alward WL, Small KW, Datson NA, Siegel-Bartelt J, Bierke-Nelson D, Bitoun P, Zabel BU, Carey JC, Murray JC (1996). Cloning and characterization of a novel bicoid-related homeobox transcription factor gene, RIEG, involved in Rieger syndrome. Nat Genet.

[B47] Furukawa T, Kozak CA, Cepko CL (1997). rax, a novel paired-type homeobox gene, shows expression in the anterior neural fold and developing retina. Proc Natl Acad Sci USA.

[B48] Brouwer A, ten Berge D, Wiegerinck R, Meijlink F (2003). The OAR/aristaless domain of the homeodomain protein Cart1 has an attenuating role in vivo. Mech Dev.

[B49] Norris RA, Kern MJ (2001). Identification of domains mediating transcription activation, repression, and inhibition in the paired-related homeobox protein, Prx2 (S8). DNA Cell Biol.

[B50] Vadlamudi U, Espinoza HM, Ganga M, Martin DM, Liu X, Engelhardt JF, Amendt BA (2005). PITX2, beta-catenin and LEF-1 interact to synergistically regulate the LEF-1 promoter. J Cell Sci.

[B51] Moede T, Leibiger B, Pour HG, Berggren P, Leibiger IB (1999). Identification of a nuclear localization signal, RRMKWKK, in the homeodomain transcription factor PDX-1. FEBS Lett.

[B52] Hessabi B, Ziegler P, Schmidt I, Hessabi C, Walther R (1999). The nuclear localization signal (NLS) of PDX-1 is part of the homeodomain and represents a novel type of NLS. Eur J Biochem.

[B53] Kozlowski K, Walter MA (2000). Variation in residual PITX2 activity underlies the phenotypic spectrum of anterior segment developmental disorders. Hum Mol Genet.

[B54] Sabherwal N, Schneider KU, Blaschke RJ, Marchini A, Rappold G (2004). Impairment of SHOX nuclear localization as a cause for Leri-Weill syndrome. J Cell Sci.

[B55] Sabherwal N, Blaschke RJ, Marchini A, Heine-Suner D, Rosell J, Ferragut J, Blum WF, Rappold G (2004). A novel point mutation A170P in the SHOX gene defines impaired nuclear translocation as a molecular cause for Leri-Weill dyschondrosteosis and Langer dysplasia. J Med Genet.

[B56] Tomura H, Nishigori H, Sho K, Yamagata K, Inoue I, Takeda J (1999). Loss-of-function and dominant-negative mechanisms associated with hepatocyte nuclear factor-1beta mutations in familial type 2 diabetes mellitus. J Biol Chem.

[B57] Griffith M, Osborne R, Munger R, Xiong X, Doillon CJ, Laycock NL, Hakim M, Song Y, Watsky MA (1999). Functional human corneal equivalents constructed from cell lines. Science.

[B58] NIH Image. http://rsb.info.nih.gov/nih-image.

